# Health risk factors in different educational groups and their association to Barrett’s esophagus

**DOI:** 10.1007/s00508-025-02575-x

**Published:** 2025-07-25

**Authors:** Nikolaus Götz, Andreas Völkerer, Hannah Hofer, Sarah Wernly, Georg Semmler, Ewald Wöll, Elmar Aigner, Maria Flamm, Christian Datz, Bernhard Wernly

**Affiliations:** 1 Department of Internal Medicine, St. Vinzenz Hospital Zams, Tyrol, Austria; 2 Paracelsus Medical University, Salzburg, Austria; 3 Department of Internal Medicine, General Hospital Oberndorf, Teaching Hospital of the Paracelsus Medical University Salzburg, Salzburg, Austria; 4 Division of Gastroenterology and Hepatology, Department of Medicine III, Medical University of Vienna, Vienna, Austria; 5 Clinic I for Internal Medicine, University Hospital Salzburg, Paracelsus Medical University, Salzburg, Austria; 6 Institute of General Practice, Family Medicine and Preventive Medicine, Center for Public Health and Healthcare Research, Paracelsus Medical University, Salzburg, Austria

**Keywords:** Chronic disease epidemiology, Lifestyle-related disorders, Esophageal cancer prevention, Esophageal neoplasms, Public health

## Abstract

**Introduction:**

General health risk factors may vary significantly across different education groups. These disparities in lifestyle choices can contribute to the development of chronic conditions, including gastrointestinal disorders. Barrett’s esophagus (BE), a premalignant condition associated with increased risk of esophageal cancer, may be influenced by these social determinants of health. This work explores how education status shapes the prevalence of BE, considering how key health risk factors in different education groups impact the development of the condition.

**Methods:**

We analyzed data from a cohort of 5160 Austrian individuals who underwent esophagogastroduodenoscopy (EGD) and screening colonoscopy. Participants were categorized into three education groups: low (*n* = 1933), medium (*n* = 2780), and high (*n* = 447). The distribution of risk factors across education groups was observed and the prevalence of BE (including any BE and dysplastic BE) was assessed using univariate and multivariable regression analyses, adjusting for potential confounders such as age, sex, metabolic syndrome, smoking, alcohol consumption, reflux severity, hiatal hernia and proton pump inhibitor intake.

**Results:**

General health risk factors, such as metabolic syndrome, alcohol consumption, gastroesophageal reflux and smoking are more prevalent in lower education groups, contributing to a higher burden of chronic diseases. The prevalence of histologically confirmed Barrett’s esophagus was low across all education levels, showing no significant differences (*p* = 0.90). Nondysplastic BE was present in 1% of participants, with similar rates across the low, medium and high education groups (1% each). Only one case of high-grade dysplasia (HGD) was found in the medium education group. In the unadjusted analysis no significant link was found between education level and Barrett’s esophagus. Compared to those with lower education, the odds were 1.25 (95% confidence interval, CI: 0.71–2.19, *p* = 0.443) for medium and 0.91 (95% CI: 0.31–2.69, *p* = 0.864) for high education.

In the fully controlled model, accounting for age, sex, metabolic syndrome, smoking, alcohol consumption, reflux severity, hiatal hernia, and proton pump inhibitor use, the association remained nonsignificant, with odds ratios of 1.15 (95% CI: 0.55–2.40, *p* = 0.719) for medium and 1.01 (95% CI: 0.30–3.36, *p* = 0.986) for high education.

**Conclusion:**

Our study indicates that education level is associated with the distribution of general risk factors, but it is not a key factor in Barrett’s esophagus risk in an asymptomatic screening population. Although education impacts health outcomes in many conditions, its influence on BE seems minimal. Future research should explore broader socioeconomic factors, such as income, occupation and healthcare access for a better understanding of the BE risk and detection disparities.

## Introduction

Education level (EduLvl) plays a crucial role in health, as individuals with lower education levels are typically more vulnerable to common health risk factors. These factors, such as smoking, poor diet, physical inactivity and alcohol consumption, are more common in lower education groups, contributing to poorer overall health outcomes and may lead to the development of chronic conditions, including gastrointestinal disorders [1–4].

Barrett’s esophagus is a premalignant condition characterized by the replacement of normal stratified squamous epithelium in the esophagus with metaplastic columnar epithelium containing goblet cells [5]. With an estimated global prevalence of 1.5%, BE is primarily associated with gastroesophageal reflux disease (GERD), which over time leads to chronic epithelial damage and, in some cases, progression to esophageal adenocarcinoma (EAC) [6]. The rising incidence of EAC, along with its poor prognosis due to late detection, underscores the importance of identifying risk factors for BE and its progression [7].

Several demographic and clinical factors are well-established risk factors for BE, including male sex, older age, smoking, central obesity and European descent [8]. The intrinsic relationship between education and general health is undeniable. Education is strongly linked to health behavior, access to medical care and chronic disease risk [9, 10]; however, the potential influence of socioeconomic status, particularly education achievement in the occurrence of BE remains unclear. Prior studies have suggested that lower education status may increase the risk of EAC but evidence regarding its association with BE remains conflicting [11–15].

Some reports have even described a paradoxical relationship in which higher socioeconomic status correlates with greater BE prevalence [16, 17], while others have found no significant association [18, 19].

Given that many established risk factors for BE, such as metabolic syndrome, smoking, and obesity, are known to vary across education strata [20, 21], we hypothesized that there might be a univariate association between education and BE; however, it remained unclear whether this effect would persist as an independent link after adjusting for known risk factors.

On an individual level, patient management could be adjusted according to educational background, with more targeted counselling on risk factors and disease progression. At a population level, prevention strategies might need to be tailored to different education groups, taking variations in health literacy, lifestyle and healthcare access into account. In an even broader sense, educational disparities in BE risk could justify distinct screening recommendations for specific subgroups, ensuring that high-risk populations are appropriately monitored and managed [22–24].

Given these potential implications, further investigation is warranted to determine whether education status influences BE risk. Our study aims to assess the prevalence of BE in a Central European screening cohort and examine its potential association with educational attainment, categorized according to the International Standard Classification of Education (ISCED) [25]. We also adjust for key demographic and metabolic risk factors, including age, sex, metabolic syndrome, smoking, alcohol consumption, reflux severity, hiatal hernia and proton pump inhibitor intake.

## Study population

This study is based on data from the Salzburg Colon Cancer Prevention Initiative (SAKKOPI), a research project screening asymptomatic individuals for colorectal cancer between January 2007 and March 2020 at a single Austrian center. The cohort included 5160 patients who underwent esophagogastroduodenoscopy as part of the screening colonoscopy. In addition to endoscopic findings, detailed medical histories, anthropometric measurements, lifestyle factors and laboratory results were collected.

### Classification of education status

Educational attainment was categorized using the Generalized International Standard Classification of Education (GISCED), a modified version of ISCED. Based on this classification, participants were divided into three educational strata: lower education (GISCED 1–2) corresponding to primary education, intermediate education (GISCED 3–4), which includes lower secondary education and vocational training and higher education (GISCED 5–6), which represents tertiary education or equivalent levels [25].

### Diagnosis of general risk factors and Barrett’s esophagus

Health-related data were measured, collected and transferred into a table for patients from the SAKKOPI cohort who came for a gastrointestinal endoscopy. These data were systematically recorded for further analysis and reference.

Barrett’s esophagus was diagnosed based on endoscopic findings, with histological confirmation used to classify cases as Barrett’s esophagus with or without dysplasia [26]. Those with suspected Barrett’s esophagus who did not have histological confirmation were considered negative.

### Statistical analysis

Continuous variables were assessed for normality and compared using either Student’s t‑test for normally distributed data or the Mann-Whitney U test for non-normally distributed data. Categorical variables were expressed as percentages and compared using the χ^2^-test.

To investigate the association between education status and the prevalence of Barrett’s esophagus, multivariable logistic regression analysis was performed, with education level as the primary predictor and the lowest education group serving as the reference category. For the analysis two models were used: the first investigated the unadjusted association between education level and Barrett’s esophagus, while the second was a fully adjusted model controlling for age, sex, metabolic syndrome, smoking, alcohol consumption, severity of reflux disease, hiatal hernia and use of proton pump inhibitors.

Results were reported as odds ratios (OR) with 95% confidence intervals (CI). Statistical significance was set at a *p*-value threshold of less than 0.05. All analyses were conducted using StataNow (StataCorpLLC, College Station, TX, USA).

## Results

### Baseline characteristics and general risk factors

A total of 5160 individuals were included in the analysis, with 1933 (37%) classified as having lower education, 2780 (54%) as having medium education, and 447 (9%) as having high education. Significant differences in baseline characteristics were observed across education groups (Table 1).

**Table 1:** Baseline characteristics of patients with lower (GISCED 1 and 2), medium (GISCED 3 and 4), and higher (GISCED 5 and 6) education. Most continuous variables were non-normally distributed. Continuous data are given as median ± interquartile range (IQR) and compared using Mann’s Whitney *U *test or mean ± standard deviation (SD) and compared using Student’s *t *test accordingly. Categorical data are given as numbers (percentage) and compared using the χ^2^-test. All tests were two-sided, and a *p*-value of < 0.05 was considered statistically significant

	Total	Low education group	Medium education group	High education group	*p*-value
	*N* = 5160	*N* = 1933	*N* = 2780	*N* = 447	
Age (years)	57 (52–65)	61 (53–68)	56 (51–63)	54 (51–60)	< 0.001
Sex (male)	52% (2707)	44% (853)	57% (1595)	58% (259)	< 0.001
Body mass index (BMI, kg/m^2^)	26 (24–30)	27 (24–30)	26 (24–29)	25 (23–28)	< 0.001
*BMI WHO categories*					< 0.001
Underweight	1% (34)	1% (15)	1% (19)	0% (0)	
Normal weight	36% (1835)	32% (611)	36% (1010)	48% (214)	
Pre-obesity	41% (2111)	41% (795)	41% (1137)	40% (179)	
Obesity	23% (1180)	26% (512)	22% (614)	12% (54)	
Waist circumference (cm)	96 (88–105)	98 (89–106)	96 (88–104)	92 (85–100)	< 0.001
Systolic blood pressure (mm Hg)	130 (120–140)	130 (120–140)	130 (120–140)	130 (120–140)	< 0.001
Diastolic blood pressure (mm Hg)	80 (75–90)	80 (80–90)	80 (75–90)	80 (74–85)	0.095
Total cholesterol (mg/dl)	220 (191–249)	219 (190–250)	220 (192–249)	219 (193–245)	0.96
HDL cholesterol (mg/dl)	56 (47–67)	57 (48–69)	55 (46–66)	55 (47–66)	< 0.001
HbA1c (%)	5.5 (5.2–5.8)	5.6 (5.4–5.9)	5.4 (5.2–5.7)	5.4 (5.2–5.6)	< 0.001
*Diabetes status*					< 0.001
No diabetes	43% (2206)	33% (646)	47% (1297)	59% (263)	
Prediabetes	44% (2257)	50% (964)	41% (1143)	34% (150)	
Diabetes	14% (697)	17% (323)	12% (340)	8% (34)	
*ATP III metabolic syndrome (≥* *3 criteria)*					< 0.001
No MetS (< 3 criteria)	13% (621)	9% (157)	14% (372)	21% (92)	
MetS (≥ 3 criteria)	87% (4207)	91% (1557)	86% (2310)	79% (340)	
*Smoking status*					< 0.001
Never smoker	40% (1735)	27% (356)	44% (1142)	54% (237)	
Ex-smoker	38% (1624)	44% (566)	36% (925)	30% (133)	
Active smoker	22% (961)	29% (374)	20% (516)	16% (71)	
*Alcohol use*					< 0.001
< 2 drinks/day	89% (4347)	80% (1408)	94% (2519)	96% (420)	
≥ 2 drinks/day	11% (544)	20% (356)	6% (169)	4% (19)	
PPI use	7% (361)	9% (179)	6% (171)	2% (11)	< 0.001
NSAID medication	1% (41)	1% (26)	1% (14)	0% (1)	0.002

Participants with lower education were older (median 61 years, IQR: 53–68 years) compared to those with medium (56 years, IQR: 51–63 years) and high education (54 years, IQR: 51–60 years, *p* < 0.001). The proportion of male participants increased with education level (44% in the lower, 57% in the medium and 58% in the high education group, *p* < 0.001).

A gradual decline in metabolic risk factors was observed with increasing education. Individuals with lower education had a higher median BMI (27 kg/m^2^, IQR: 24–30 kg/m^2^) compared to the medium (26 kg/m^2^, IQR: 24–29 kg/m^2^) and high education groups (25 kg/m^2^, IQR: 23–28 kg/m^2^, *p* < 0.001). Similarly, waist circumference was highest in the lower education group (98 cm, IQR: 89–106 cm) compared to the medium (96 cm, IQR: 88–104 cm) and high education group (92 cm, IQR: 85–100 cm, *p* < 0.001).

**Fig. 1:**
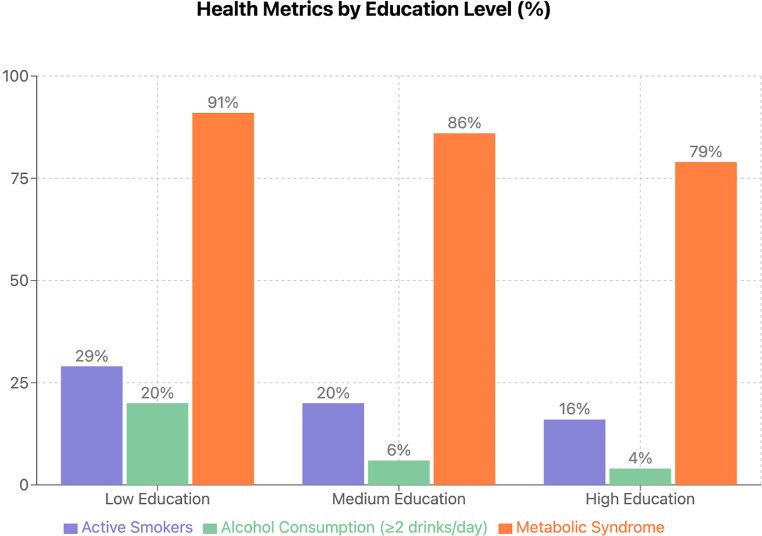
Prevalence of behavioral and metabolic risk factors by education level. Bar graph showing the percentage prevalence of active smoking, alcohol consumption (≥ 2 drinks/day), and metabolic syndrome stratified by education level (low, medium, high). All three risk factors demonstrated statistically significant inverse associations with education level (*p* < 0.001 for all comparisons). Metabolic syndrome showed the highest overall prevalence, affecting 91%, 86%, and 79% of individuals in the low, medium, and high education groups, respectively. Similarly, active smoking (29%, 20%, 16%, respectively) and alcohol consumption of ≥ 2 drinks/day (20%, 6%, 4%, respectively) were most prevalent in the low education group. These findings suggest a consistent socioeconomic gradient in health risk behavior and metabolic outcomes

Lifestyle factors also varied significantly (Fig. 1). Smoking prevalence was highest in the lower education group, with 29% active smokers, compared to 20% in the medium and 16% in the high education group (*p* < 0.001). Additionally, alcohol consumption of ≥ 2 drinks/day was more common in individuals with lower education (20% vs. 6% vs. 4%, *p* < 0.001, respectively). The prevalence of metabolic syndrome (MetS) was highest among those with lower education (91% vs. 86% vs. 79%, *p* < 0.001, respectively). Diabetes prevalence decreased with increasing education (17% in the lower, 12% in the medium, and 8% in the high education group, *p* < 0.001).

Participants with lower education were also more likely to have prediabetes (50% vs. 41% vs. 34%, *p* < 0.001, respectively). The use of PPI was significantly higher in the lower education group (9% vs. 6% vs. 2%, *p* < 0.001, respectively), while NSAID use remained low across all groups (1%, *p* = 0.002).

### Educational level and gastroesophageal findings

Significant differences in gastroesophageal conditions were observed across education levels (Table 2).

**Table 2:** Gastroesophageal findings in different education levels

	Total	Low education group	Medium education group	High education group	*p*-value
	*N* = 5160	*N* = 1933	*N* = 2780	*N* = 447	
Hiatal hernia	53% (2747)	58% (1112)	51% (1411)	50% (224)	< 0.001
*Reflux esophagitis (LA cassification)*					< 0.001
Grade 0	61% (3119)	61% (1177)	60% (1671)	61% (271)	
Grade A	32% (1667)	36% (697)	30% (842)	29% (128)	
Grade B	5% (233)	2% (35)	6% (163)	8% (35)	
Grade C	2% (94)	1% (10)	3% (74)	2% (10)	
Grade D	0% (22)	0% (2)	1% (18)	0% (2)	
*Histological Barrett’s esophagus*					0.90
No Barrett	99% (5103)	99% (1914)	99% (2746)	99% (443)	
Non-dysplastic Barrett	1% (55)	1% (19)	1% (32)	1% (4)	
Low-grade dysplasia	0% (1)	0% (0)	0% (1)	0% (0)	
High-grade dysplasia	0% (1)	0% (0)	0% (1)	0% (0)	

Hiatal hernia prevalence was highest in individuals with lower education (58%), compared to 51% in the medium and 50% in the high education group (*p* < 0.001).

Similarly, the prevalence of reflux esophagitis (LA classification A–D) was higher among individuals with lower education. While 61% of all participants had no signs of esophagitis, those with lower education had a higher prevalence of grade A esophagitis (36%), compared to 30% in the medium and 29% in the high education group. In contrast, higher grades of esophagitis (B–D) were slightly more frequent in the medium and high education groups, although absolute numbers remained low (*p* < 0.001).

### Prevalence of Barrett’s esophagus

The prevalence of histologically confirmed Barrett’s esophagus was low across all education groups, with no significant differences (*p* = 0.90). Nondysplastic Barrett’s esophagus was detected in 1% of all participants, with comparable proportions across the lower (1%), medium (1%), and high (1%) education groups. Only one case of high-grade dysplasia was identified in the medium education group. In the unadjusted model (model 1), we observed no statistically significant association between education level and Barrett’s esophagus. Compared to participants with lower education, those with medium education had an odds ratio (OR) of 1.25 (95% CI: 0.71–2.19, *p* = 0.443) and those with high education had an OR of 0.91 (95% CI: 0.31–2.69, *p* = 0.864).

In the fully adjusted model (model 2, Table 3), which is controlled for age, sex, metabolic syndrome, nicotine and alcohol consumption, reflux disease severity, hiatal hernia and proton pump inhibitor use, the association between education level and Barrett’s esophagus remained nonsignificant, with ORs of 1.15 (95% CI: 0.55–2.40, *p* = 0.719) for medium education and 1.01 (95% CI: 0.30–3.36, *p* = 0.986) for high education.

**Table 3:** Model 2—Barrett’s esophagus and education level fully adjusted model controlled for age, sex, metabolic syndrome, nicotine and alcohol consumption, reflux disease severity, hiatal hernia, and proton pump inhibitor use compared to lower education level.

	Odds ratio (OR)	95% confidence interval (CI)	*p*-value
Medium education group	1.15	0.55–2.40	0.72
High education group	1.01	0.30–3.36	0.99
Age	1.00	0.90–0.97	0.90
Male sex	2.83	0.01–1.29	0.01
MetS (> 3 criteria)	2.30	0.54–9.80	0.26
Ex-smoker	1.39	0.67–2.90	0.37
Active smoker	1.73	0.76–3.96	0.19
Alcohol consumption (> 2 drinks/day)	0.03	0.35–2.50	0.90
GERD A	2.11	1.06–4.24	0.03
GERD B	2.58	0.90–7.42	0.08
GERD C	5.29	1.64–17.06	0.01
Hiatal hernia	1.94	0.95–3.40	0.07
PPI use	1.66	0.63–4.35	0.30

## Discussion

In this large population-based study, we investigated the relationship between education level and common health-related determinants. Additionally, we examined the potential association between education status and the risk of developing Barrett’s esophagus, while adjusting for key risk factors such as age, sex, metabolic syndrome, smoking, alcohol consumption, reflux severity, hiatal hernia and proton pump inhibitor intake.

Our findings imply that individuals with higher educational attainment tend to engage in healthier behavior, such as regular physical activity and healthier dietary choices, compared to those with lower levels of education. This aligns with the existing literature, which consistently highlights the link between education and various health determinants, including access to healthcare, health literacy and lifestyle choices [27–29]; however, it is important to note that while education is a strong predictor of health behavior, other socioeconomic factors, such as income and employment, also significantly contribute to health disparities.

In many of the available studies, the abovementioned influences are considered to be risk factors for the occurrence of Barrett’s esophagus [30–36].

The results suggest that educational attainment by itself does not have a major impact on the development of BE in asymptomatic screening populations. A low prevalence of dysplastic BE across all education groups indicates that other factors, more directly related to the disease’s pathogenesis, may require further investigation.

The relationship between socioeconomic status, including educational attainment, and BE risk remains poorly understood, with previous studies yielding inconsistent results. Our study aligns with research that has failed to establish a clear and consistent connection between education status and BE risk; however, these findings contrast with studies that propose a paradoxical association, where higher levels of education are linked to increased BE prevalence [16, 17, 37, 38]. On the other hand, studies have suggested that lower socioeconomic status correlates with higher rates of esophageal adenocarcinoma, which could be indirectly associated with BE [39]. There are also studies with the result of no association between socioeconomic or education status and the occurrence of BE [18, 19].

The inconsistencies across studies may stem from differences in study populations, geographic regions, and methods used to define socioeconomic status. Unmeasured confounders, such as healthcare access, dietary habits, or regional lifestyle variations, could also influence these relationships and contribute to the contradictions in the literature.

Our study highlights the complexity of interpreting the role of socioeconomic factors, like education, in BE development. While education itself may not directly influence BE risk, factors like healthcare access, health literacy, and lifestyle choices likely mediate the relationship. Higher education may lead to better health behavior, earlier interventions and reduced risk from factors like obesity and smoking. Individuals with higher socioeconomic status may be more likely to participate in preventive screening [40, 41]. This increased participation could potentially inflate the observed prevalence of BE within this group.

A key finding in our study was the low prevalence of BE across all education groups, suggesting that other risk factors, such as gastroesophageal reflux disease, obesity, and genetic predisposition, may have a more significant impact on BE development than education status. The presence of GERD, in particular, is the most well-established risk factor for BE and individuals with symptomatic GERD are more likely to seek medical attention and undergo screening, increasing their likelihood of a BE diagnosis. This may partly explain the low prevalence of BE in our cohort, as individuals with asymptomatic GERD may be underdiagnosed and less likely to participate in screening. Additionally, BE’s multifactorial nature implies that genetic, environmental and behavioral factors contribute to its development. Our study suggests that BE risk results from complex interactions among these factors, with education status influencing healthcare access and engagement in screening, rather than directly determining BE risk.

An intriguing finding of our analysis is the higher prevalence of known risk factors in groups with lower educational attainment, despite the absence of a corresponding increase in the prevalence of Barrett’s esophagus. This discrepancy suggests that while lower education may be linked to lifestyle or environmental factors that generally elevate health risks, such as obesity, smoking and poor diet, these do not necessarily translate into a higher incidence of BE in asymptomatic populations. Possible explanations include the multifactorial nature of BE development, where genetic predisposition, reflux severity, and other biological factors play significant roles that may offset or modulate the impact of socioeconomic risk factors.

The higher prevalence of hiatal hernia in individuals with lower educational attainment may reflect a combination of lifestyle, occupational and healthcare-related factors. People with lower education levels are more likely to be obese and engage in behavior, such as smoking and poor diet, which increase intra-abdominal pressure. They may also be employed in physically demanding jobs involving heavy lifting, a known risk factor for hernia formation. Limited health literacy could delay symptom recognition and reduce engagement with preventive healthcare. Additionally, higher levels of chronic stress and comorbid conditions like GERD or chronic cough may further contribute to hernia development. These interconnected factors suggest that the association is likely multifactorial rather than directly caused by education level alone.

Higher proton pump inhibitor use and greater GERD prevalence were observed in lower education groups, yet this did not translate into higher rates of Barrett’s esophagus. This finding may suggest a potential protective effect of proton pump inhibitors in preventing the progression from GERD to BE. Additionally, early diagnosis and management of GERD could be more common or effective in these groups, mitigating BE risk; however, cross-sectional data limit causal interpretation and longitudinal studies are needed to clarify these relationships over time. Further investigation should focus on long-term outcomes to better understand how PPI use and GERD management influence BE development.

While our research benefits from a large sample size and the inclusion of histologically confirmed BE diagnoses, several important limitations must be considered.

First, the cross-sectional nature of our study precludes any conclusions regarding causality. It is possible that other factors, such as lifestyle changes or interventions that occur after the diagnosis of BE, may influence the observed associations. Longitudinal cohort studies that follow participants over time would be invaluable in establishing the temporal relationship between educational attainment, lifestyle factors, and the development of BE.

The study was conducted in a single geographic region, which may limit the generalizability of the findings to other populations with different healthcare access, lifestyle practices, or genetic predispositions. Regional variations in healthcare infrastructure, dietary habits, and environmental exposures could play a crucial role in the development of BE. Multicenter studies that incorporate diverse populations from various regions would provide a more comprehensive understanding of the relationship between education status and BE risk.

Selection bias is an important consideration. Our cohort consisted of individuals who were part of a screening program, and these participants may differ systematically from the general population in ways that are not accounted for in the analysis. For example, individuals who are more health-conscious or have higher health literacy may be more likely to engage in screening programs, leading to an overrepresentation of certain socioeconomic groups. This self-selection bias could skew the results, particularly if those with higher education levels are more likely to be aware of BE risk factors and engage in screening early.

Our dataset lacked detailed socioeconomic variables such as income, occupation, and comprehensive medical histories, including GERD severity, which may affect BE risk and explain discrepancies with previous studies.

## Conclusion

In conclusion, our study suggests that although a low education level may be associated with limited knowledge about healthy lifestyles and preventive care, it was not shown to be an independent health risk factor and education status did not significantly influence the risk of Barrett’s esophagus in asymptomatic screening populations. While the literature on socioeconomic status and BE is inconsistent, our findings contribute to the growing body of evidence suggesting that BE risk is influenced by a complex interplay of behavioral, genetic and environmental factors rather than by educational attainment alone. Future research should adopt a broader, more nuanced approach, incorporating multiple dimensions of the socioeconomic status and considering additional factors such as healthcare access, lifestyle behavior and genetic predisposition. This will help further clarify the role of socioeconomic factors in the development of BE and provide information on targeted prevention strategies to mitigate risk in diverse populations.

## Data Availability

The data associated with this submission can be made available upon reasonable request.
